# Anterior-enriched filopodia create the appearance of asymmetric membrane microdomains in polarizing *C. elegans* zygotes

**DOI:** 10.1242/jcs.230714

**Published:** 2019-07-15

**Authors:** Nisha Hirani, Rukshala Illukkumbura, Tom Bland, Grégoire Mathonnet, Delphine Suhner, Anne-Cecile Reymann, Nathan W. Goehring

**Affiliations:** 1 The Francis Crick Institute, 1 Midland Road, London NW1 1AT, UK; 2 Institute for the Physics of Living Systems, University College London, London WC1E 6BT, UK; 3 Institut de Génétique et de Biologie Moléculaire et Cellulaire, Centre National de la Recherche Scientifique, UMR7104, Institut National de la Santé et de la Recherche Médicale, U1258, and Université de Strasbourg, 67404 Illkirch, France; 4 MRC Laboratory for Molecular Cell Biology, University College London, Gower Street, London WC1E 6BT, UK

**Keywords:** *C. elegans*, PAR proteins, PIP_2_, Cell cortex, Cell polarity, Filopodia

## Abstract

The association of molecules within membrane microdomains is critical for the intracellular organization of cells. During polarization of the *C. elegans* zygote, both polarity proteins and actomyosin regulators associate within dynamic membrane-associated foci. Recently, a novel class of asymmetric membrane-associated structures was described that appeared to be enriched in phosphatidylinositol 4,5-bisphosphate (PIP_2_), suggesting that PIP_2_ domains could constitute signaling hubs to promote cell polarization and actin nucleation. Here, we probe the nature of these domains using a variety of membrane- and actin cortex-associated probes. These data demonstrate that these domains are filopodia, which are stimulated transiently during polarity establishment and accumulate in the zygote anterior. The resulting membrane protrusions create local membrane topology that quantitatively accounts for observed local increases in the fluorescence signal of membrane-associated molecules, suggesting molecules are not selectively enriched in these domains relative to bulk membrane and that the PIP_2_ pool as revealed by PH_PLCδ1_ simply reflects plasma membrane localization. Given the ubiquity of 3D membrane structures in cells, including filopodia, microvilli and membrane folds, similar caveats are likely to apply to analysis of membrane-associated molecules in a broad range of systems.

## Abstract (precis)

**Summary:** Apparent accumulation of PIP_2_ and cortex/polarity-related proteins within plasma membrane microdomains in polarizing *C. elegans* zygotes reflects local membrane topology induced by filopodia, not selective enrichment within signaling domains.

## INTRODUCTION

Micro- to nano-scale heterogeneity in the distribution of proteins and lipids in the plasma membrane has emerged as a fundamental organizing principle of the cell (Simons and Ikonen, 1997; Balla, 2013; Schink et al., 2016; Stone et al., 2017). By partitioning molecules into distinct compartments, local clustering can also serve a potentially powerful mechanism for regulating molecular behavior.

**Fig. 1.**
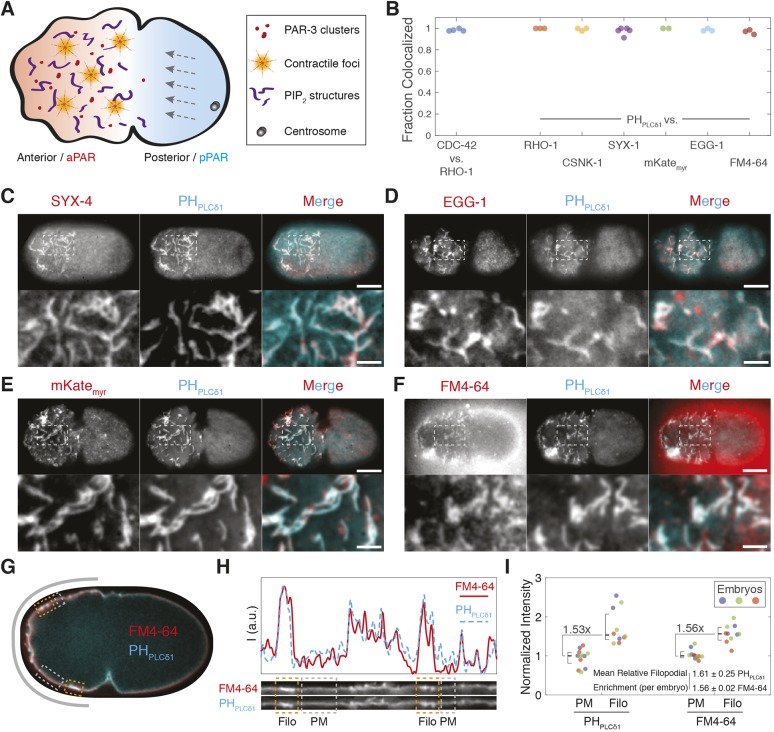
**Diverse membrane-associated molecules co-label common membrane structures.** (A) Schematic of *C. elegans* zygote polarization, highlighting PAR-3 clusters, contractile foci and putative PIP_2_-enriched membrane domains. Polarization of PAR proteins (red–blue) is induced by anterior-directed actomyosin cortical flows (gray arrows). (B) Fraction of membrane structures co-labeled by the indicated markers. Sample images are shown in C–F and Fig. S2. (C–F) Surface images of embryos expressing fluorescent protein (FP)-tagged PH_PLCδ1_ with transmembrane syntaxin, SYX-4 (GFP::SYX-4) (C), the oocyte-enriched membrane protein EGG-1 (GFP::EGG-1) (D), a myristoylated form of mKate, mKate_myr_ (E) and the membrane dye FM4-64 (F). Individual channels and merged images are shown (whole embryo and a magnification of the indicated area). Scale bars: 10 µm (embryo), 2.5 µm (magnification). (G) Cross-section of embryo expressing GFP-tagged PH_PLCδ1_ with FM4-64 (*n*=3). Boxes highlight plasma membrane (gray, PM) and filopodia (orange, Filo). The gray band indicates the region straightened in H. (H) A 20-pixel-wide straightened region taken along the indicated path in G. PH_PLCδ1_ and FM4-64 intensity are shown individually with intensity plots (a.u., arbitrary units) above. Orange and gray boxes highlight regions marked in G. (I) Fluorescence intensity for PM and Filo regions from embryos co-labeled with PH_PLCδ1_ and FM4-64. Intensity normalized to embryo median (set as 1, color coded) with overall median±95% c.i. shown for all datapoints. Relative median filopodia enrichment between probes is indicated. The relative filopodia enrichment for individual embryo means is also provided (mean±s.d.).

During polarity establishment in the *C. elegans* zygote, clustering of a conserved set of PAR proteins (PAR-3, PAR-6 and PKC-3) on the membrane is critical for their ability to be segregated into the nascent anterior by actomyosin cortical flows (Rodriguez et al., 2017; Wang et al., 2017; Dickinson et al., 2017), eventually allowing them to be replaced by a second opposing set of PAR proteins (PAR-1, PAR-2, LGL-1 and CHIN-1) on the posterior membrane (Rose and Gonczy, 2014; Goehring, 2014). Cortical flows are in turn controlled by local foci of RHO-1 activation, which drive pulsatile actin nucleation and contraction of the cortical actomyosin network (Nishikawa et al., 2017; Michaux et al., 2018) (summarized in Fig. 1A).

Asymmetric enrichment of phosphatidylinositol 4,5-bisphosphate (PIP_2_), has been observed within another class of membrane-associated domains in the anterior of the *C. elegans* zygote (Nakayama et al., 2009; Wang et al., 2017; Scholze et al., 2018). Similar enrichment is seen for the polarity-related Rho-family GTPases CDC-42 and RHO-1, the RHO-1 regulator ECT-2, a CDC-42-associated sub-population of PAR-6 and PKC-3, and casein kinase (CSNK-1) (Motegi and Sugimoto, 2006; Schonegg et al., 2007; Panbianco et al., 2008; Wang et al., 2017). PIP_2_-enriched microdomains have been proposed to serve as organizing platforms to coordinate regulation of cortical actin organization, cell polarity and asymmetric division of the zygote (Scholze et al., 2018). Despite being noted over a decade ago, the nature of these domains remains poorly understood. Here, we show that these apparent microdomains are filopodia, which create the illusion of local enrichment of membrane-associated molecules due to induction of changes in local membrane topology. Our data argues against local enrichment of PIP_2_ within the anterior of the embryo or within micron-scale domains.

## RESULTS AND DISCUSSION

### Diverse membrane-associated molecules appear to be co-enriched in membrane structures

To reveal the nature of these PIP_2_-enriched domains, we confirmed previous results that polarity-related proteins RHO-1, CDC-42, and CSNK-1 colocalized to a similar class of membrane-associated domains labeled by the PIP_2_ probe, PH_PLCδ1_ in *C. elegans* zygotes. All proteins labeled similar domains, which varied with the cell cycle, peaked during polarity establishment and colocalized with >90% of PIP_2_-labeled domains (Nakayama et al., 2009; Motegi and Sugimoto, 2006; Schonegg et al., 2007; Panbianco et al., 2008; Scholze et al., 2018) (Fig. 1B; Figs S1 and S2). Given this coincidence, we determined whether the co-labeling was specific. We therefore co-expressed PH_PLCδ1_ with various plasma membrane markers, including the syntaxin SYX-4 (Jantsch-Plunger and Glotzer, 1999), a myristoylated form of mKate, mKate_myr_, and the plasma membrane protein EGG-1 (Kadandale et al., 2005). Surprisingly, all proteins marked >90% of PH_PLCδ1_-labeled domains (Fig. 1B–E). To further control for non-specific labeling of bulk plasma membrane, we examined localization of the membrane dye FM4-64, which also labeled >90% of PH_PLCδ1_-positive domains (Fig. 1B,F; Movie 1). We observed quantitative agreement in the relative enrichment of the PH_PLCδ1_ and FM4-64 signal within the domains, indicating that there was no selective enrichment of molecules, including of PIP_2_, within these domains relative to what is seen for bulk membrane (Fig. 1G–I).

### Polarizing embryos exhibit asymmetric filopodia-like structures

**Fig. 2.**
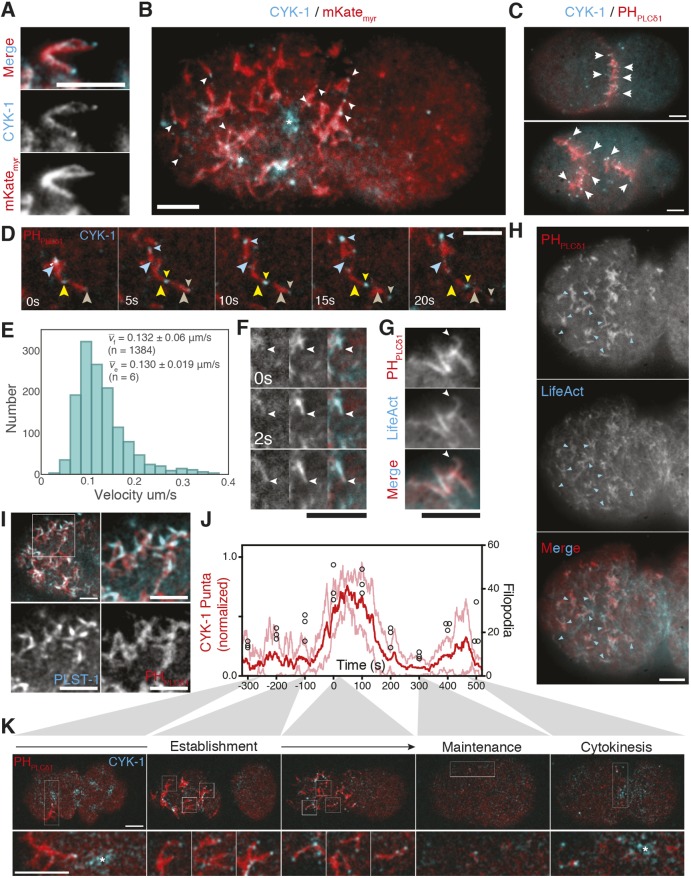
**Asymmetric cortical structures resemble filopodia.** (A) Magnified region from the pseudocleavage furrow in embryo expressing mKate_myr_ and CYK-1::GFP. See Movie 2. (B) CYK-1 localization to large contractile foci (*), and tips of filopodia-like structures revealed by mKate_myr_ (arrowheads) in embryo anterior. (C) Surface images of two- and four-cell embryos expressing CYK-1::GFP and mCherry::PH_PLCδ1_. Arrowheads mark CYK-1-tipped membrane structures at cell contacts. (D) Time course of filopodia movement. Large arrowheads denote CYK-1::GFP puncta at time=0 s. Position in subsequent frames marked by small arrowheads of corresponding color. See Movie 3. (E) Histogram of CYK-1::GFP puncta velocities. *v*_f_ denotes mean±s.d. velocity for all CYK-1 puncta. *v*_e_ is the mean of embryo means. (F) Growth time course of a single filopodium (arrowhead) from embryo shown in H highlighting LifeAct localization throughout the extending structure (mCherry::PH_PLCδ1_, left; LifeAct::GFP, middle; merge, right). See Movie 4. (G) Example of LifeAct::GFP labeling throughout extended mCherry::PH_PLCδ1_-positive filopodia (arrowhead). Labeling observed in 13/13 extended filopodia (three embryos). (H) Extensive colocalization of LifeAct::GFP with mCherry::PH_PLCδ1_ within putative filopodia (arrowheads) (*n*=4). (I) Cortical image of embryo anterior showing mCherry::PH_PLCδ1_ and PLST-1::GFP (top left). The boxed area is magnified (top right) with individual channels shown below. Quantification and additional images are in Fig. S3A,B. (J) Number of CYK-1 puncta (normalized to peak number; mean in red with *±*s.d. in pink) and PH_PLCδ1_-labeled structures (black circles) over time. Time 0 s is the transition between establishment and maintenance phase marked by relaxation of the pseudocleavage furrow. Cytokinesis occurs at between 400 and 500 s. (K) Confocal cortical images of embyro expressing CYK-1::GFP and mCherry::PH_PLCδ1_ at representative time points. Boxed areas are shown magnified 3× below. The asterisk indicates large pulsatile foci common at polarity establishment and cytokinesis. See Movie 5. Colocalization of CYK-1 puncta with mCherry::PH_PLCδ1_-labeled structures in Fig. S3A. Scale bars: 5 µm.

What could be the origin of these PH_PLCδ1_-labeled domains that could explain their non-specific labeling by membrane-associated molecules? A clue came from observations of extended tubular structures protruding from the cell that were evocative of filopodia, which were particularly evident near the pseudocleavage furrow where the membrane pulls away from the eggshell (Fig. 2A).

Filopodia are thin, dynamic, actin-rich membrane protrusions. Their formation and extension is driven by actin polymerization downstream of Arp2/3 and formins, and is regulated by actin regulatory molecules including actin-bundling and -capping proteins (Mattila and Lappalainen, 2008), with Myosin-X and formins typically enriched at their tips (Jacquemet et al., 2019). Because there is no homolog of Myosin-X in *C. elegans*, we examined localization of the embryonically expressed formin CYK-1 co-expressed with red fluorophore fusions to PH_PLCδ1_ or mKate_myr_ (Swan et al., 1998). CYK-1 was enriched at the tips of extended tubular structures and comet-like structures at the cortex (Fig. 2A,B; Movies 2 and 3). We interpret the latter structures to be the same as extended tubular structures but pressed against the embryo surface by the eggshell. Intercalating CYK-1-tipped finger-like projections were also observed at regions of cell–cell contacts at both two- and four-cell stages (Fig. 2C). CYK-1 puncta were distinct from large pulsatile foci that are also present during the polarity establishment phase (Fig. 2B,K, asterisks) and which have been shown to coincide with pulsatile actomyosin (Michaux et al., 2018).

CYK-1-tipped structures were dynamic, exhibiting processive motion across the plasma membrane at velocities consistent with prior quantification of filopodia growth rates (Argiro et al., 1985) (Fig. 2D,E). To further establish the filopodia-like nature of these structures, we examined LifeAct::GFP, which extensively colocalized with the mCherry:: PH_PLCδ1_ signal in putative filopodia and appeared to extend throughout filopodia-like structures (Fig. 2F–H, Movie 4). We also found that >80% of filopodia-like structures were labeled by the *C. elegans* ortholog of the actin-bundling protein plastin (PLST-1 in *C. elegans*; Ding et al. 2017) (Fig. 2I, Fig. S3A,B), consistent with data from other systems (Jacquemet et al., 2019). Finally, the combined loss of both CYK-1 and ARP-2/3 function prevented their formation (Fig. S3C), consistent with prior work demonstrating the dependence of PIP_2_ domains on actin (Scholze et al., 2018). By contrast, loss of either cortical contractility or PAR polarity did not affect the formation of filopodia, only their asymmetry along the anterior–posterior axis (Fig. S3D).

Numbers of CYK-1 puncta generally correlated with appearance of PH_PLCδ1_-labeled structures (Fig. 2J,K; Movie 5): Numbers of both were initially low, peaking after the transition to maintenance phase, which coincides with reorganization of the actin cortex (Fig. 2J,K, 0 s) (Munro et al., 2004; Velarde et al., 2007). Both then declined and remained largely absent until reappearing at the onset of cytokinesis (Fig. 2J,K, 400–500 s). This correlation suggests that filopodia account for the vast majority of PH_PLCδ1_-labeled structures in the zygote.

### Preferential labeling of distinct F-actin populations by different LifeAct probes

**Fig. 3.**
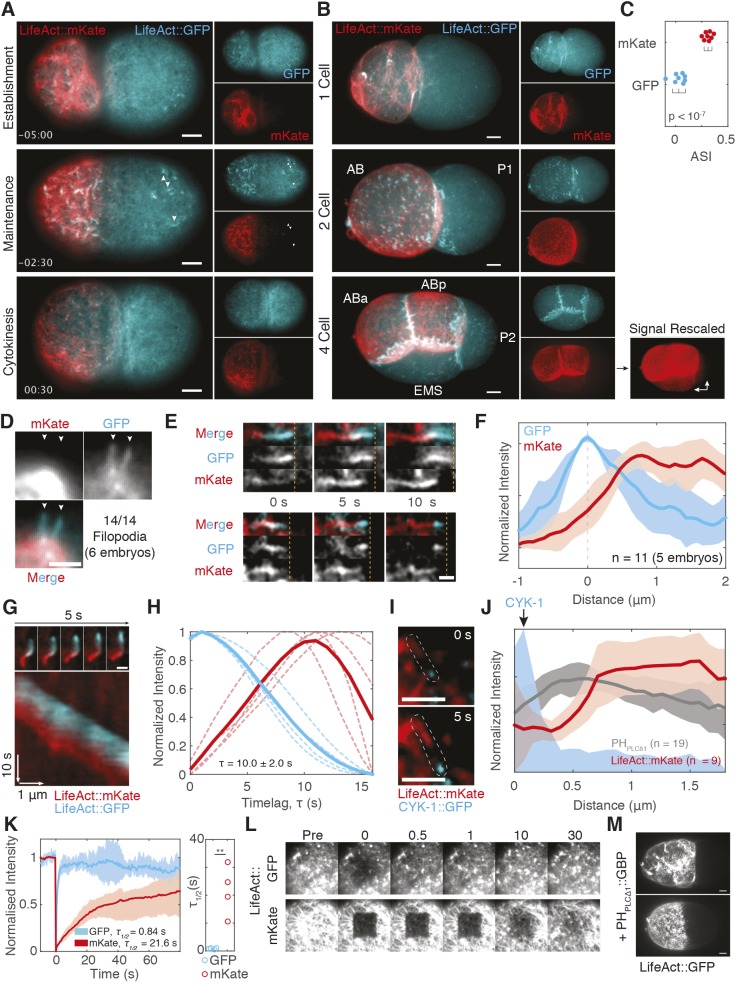
**LifeAct::GFP and LifeAct::mKate label distinct actin populations *in vivo*.** (A) Cortical images of LifeAct::mKate vs LifeAct::GFP during the first cell cycle, quantified in (C). Arrowheads mark posterior filopodial structures that are only labeled by LifeAct::GFP. Time (min:sec) relative to cytokinesis. (B) Max 3D projections of 1-, 2- and 4-cell embryos. LifeAct::mKate signal in the 4-cell embryo is shown rescaled to highlight asymmetry between EMS and P2 (arrows). (C) Asymmetry (ASI) of LifeAct::GFP vs LifeAct::mKate signal in 1-cell establishment phase embryos (panel A). (D) LifeAct::GFP, but not LifeAct::mKate, labels filopodia extending from the cell surface. (E) LifeAct::mKate lags LifeAct::GFP labeling of two processive surface-associated filopodia. Computationally straightened images shown. Dashed lines mark leading edge of GFP signal for reference. See Movie 6. (F) Lag of LifeAct::mKate relative to peak LifeAct::GFP signal in fluorescence intensity traces along filopodia. (G) Time lapse images of a cytoplasmic actin comet labeled with LifeAct::mKate and LifeAct::GFP and an associated kymograph taken along a trace of the comet path. See Movie 7. (H) Quantification of LifeAct::mKate time lag measured from kymographs as in G. Average temporal change across a minimum of ten positions for each individual comet (dashed lines, *n*=4) shown along with mean of embryo means (solid lines). Δτ is the peak-to-peak time lag. (I) Time lapse of images of a filopodium (outlined) labeled by CYK-1::GFP and LifeAct::mKate. (J) Quantification of LifeAct::mKate or mCherry::PH_PLCδ1_ relative to GFP::CYK-1 puncta. Mean±s.d. shown. (K) FRAP analysis of cortical LifeAct::GFP versus LifeAct::mKate following bleaching of a 6.2×6.2 µm box. Mean FRAP trace (±max/min; shaded area) (left) shown along with *τ*_1/2_ for each replicate. **P<0.01 (two-tailed *t*-test). (L) Time series of FRAP experiments from K. (M) Stabilization of LifeAct::GFP by membrane-tethered GFP nanobody (PH_PLCδ1_::GBP)-induced segregation. Maximum *z*-projections at establishment (top) and maintenance phase (bottom) are shown (*n*=3). Scale bars: 5 µm (A,B,D,M), 2.5 µm (E,G,I).

The colocalization we observe between LifeAct::GFP and PH_PLCδ1_ differed from that described in previous work in which PIP_2_ enrichment was reported to precede LifeAct::mKate enrichment by nearly 10 s (Scholze et al., 2018). We wondered whether the divergent results were due to employment of differently tagged versions of LifeAct. Co-expression of both GFP and mKate versions of LifeAct in embryos revealed distinct localization behaviors. Most noticeably, LifeAct::mKate appeared to segregate preferentially into the anterior (Fig. 3A,C) and was unequally inherited by the anterior daughter cell (AB) relative to its sister P1, and again by the P1 daughter EMS relative to its sister P2 (Fig. 3B). Neither behavior was observed for LifeAct::GFP. LifeAct::mKate also poorly labeled posterior structures that were labeled efficiently by LifeAct::GFP (Fig. 3A).

LifeAct::GFP and LifeAct::mKate also showed distinct labeling of filopodia. Whereas LifeAct::GFP efficiently labeled dynamic filopodia extending from the cell, LifeAct::mKate was depleted (Fig. 3D). LifeAct::mKate signal also lagged behind LifeAct::GFP signal in filopodia moving along the embryo surface and in cytoplasmic actin comets (Fig. 3E–H, Movies 6 and 7). Finally, we observed a spatial gap between CYK-1 puncta at filopodia tips and LifeAct::mKate signal, consistent with a time lag in labeling filopodia (Fig. 3I,J).

Lags in actin probe localization have been associated with slow turnover rates in the context of actin flow (e.g. for LifeAct versus utrophin; Bement et al., 2015; Maiuri et al., 2015). LifeAct is generally thought to turn over rapidly, but behavior can vary with fluorophore and expression level (Riedl et al., 2008; Spracklen et al., 2014; Courtemanche et al., 2016; van der Honing et al., 2011). We therefore performed fluorescence recovery after photobleaching (FRAP) assays to analyze the binding kinetics (Fig. 3K,L). LifeAct::mKate turnover rates were an order of magnitude slower than for LifeAct::GFP (*r*_1/2_=21*.*6±8*.*9 versus 0*.*84±0*.*27 s; mean*±*s.d.), reaching time scales comparable to turnover of cortical F-actin in the *C. elegans* cortex (Robin et al., 2014). We conclude that slow turnover of LifeAct::mKate leads to its localization to a discrete, potentially more-stable or long-lived, sub-population of actin structures, which explains the previously observed lag in LifeAct::mKate localization to PIP_2_-labeled structures (Scholze et al., 2018). The temporal lag we observe matches the reported delay between PH_PLCδ1_ and LifeAct::mKate (10 versus 9.3 s). Consistent with this interpretation, artificially stabilizing LifeAct::GFP at the membrane by co-expression with a membrane-tethered GFP-binding protein induced segregation of LifeAct::GFP, reproducing the segregation phenotype observed with LifeAct::mKate (Fig. 3M). Affinity differences in LifeAct probes could also potentially explain reported resistance of cortical actin to actin-disrupting agents in LifeAct::mKate-expressing lines relative to prior work (Goehring et al., 2011; Michaux et al., 2018; Scholze et al., 2018).

### Membrane topology quantitatively accounts for local ‘enrichment’ of membrane-associated molecules

We next sought to determine how filopodia could result in apparent local enrichment of membrane-associated molecules. One possibility is that enrichment simply reflects the local accumulation of membrane within ruffles, tubes or folds within the imaging plane, increasing local fluorescence above that seen for the surrounding single membrane bilayer. This effect, described previously in mammalian cells, would occur even if protein concentration on the membrane was uniform (van Rheenen and Jalink, 2002).

**Fig. 4.**
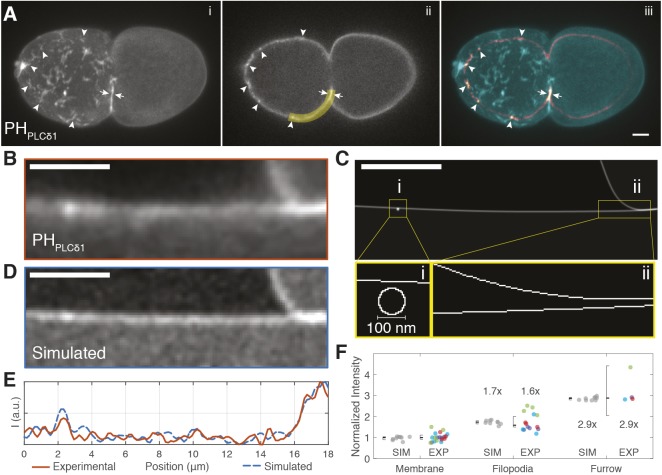
**Bulk membrane accumulation quantitatively accounts for observed cortical ‘enrichment’.** (A) Maximum *z*-projection (i), single plane (ii) and overlay (iii) of an establishment phase embryo expressing mCherry::PH_PLCδ1_. Arrowheads mark visible accumulations of signal in a single plane that can be identified as cross-sections of membrane structures based on the *z*-projection. White arrows mark double membrane generated at the pseudocleavage furrow. (B) Straightened cortical region of the experimental image taken along the yellow line in (Aii). (C) A 5 nm/pixel representation of our filopodia model of the image in B, including a 100-nm-diameter membrane tube (zoom, i) and a double membrane region at right (zoom, ii). (D) Simulated image following convolution of C. (E) Plot of mean-normalized intensity along the membrane in the experimental (B, red) and simulated image (D, dashed blue). (F) Quantification of fluorescence intensity of putative filopodia relative to single membranes and furrow regions in experimental and simulated images. Datapoints from individual embryos are color coded (*n*=4), normalized to median values and shown alongside median-normalized data from simulated image replicates (*n*=10). The median±95% c.i. is indicated by the whisker plot along with fold-change from median (set at 1). Scale bars: 5 µm.

To determine whether locally increased signal could be explained by membrane topology, we compared the distribution of fluorescence of mCherry::PH_PLCδ1_ obtained by confocal microscopy with what would be expected if membrane concentration were uniform, but a filopodia was immediately adjacent to the membrane. To this end, we obtained *z*-stacks of embryos expressing mCherry::PH_PLCδ1_ during the establishment phase. Bright spots were visible in individual planes which could be assigned to filopodia in 3-D renderings (Fig. 4A, arrowheads). These filopodia were brighter than regions containing a single membrane bilayer, but less bright than the double membrane bilayer of the pseudocleavage furrow (Fig. 4A, arrows). Quantification of experimental intensities were then compared to those obtained from a simulated image, which was constructed by assuming the presence of a single 5-nm-thick bilayer, flanked by a second bilayer in the region of the pseudocleavage furrow, and a 100-nm diameter filopodium, assuming uniform membrane concentration (Fig. 4C,D, see Materials and Methods). Intensity distributions were remarkably similar, with experimental measurements almost exactly matching predictions from simulated images (Fig. 4E,F).

Thus, for the molecules analyzed here, including PIP_2_, RHO-1, CDC-42 and CSNK-1, local cortical signal in filopodia-like structures can be fully explained by changes in local membrane topology, arguing against any concentration of these molecules within micron-scale domains in the plasma membrane or asymmetric enrichment of PIP_2_ in the zygote anterior. While filopodia are the dominant features underlying this phenomenon in the zygote, any local changes in membrane topology would give a similar appearance of local enrichment of membrane-associated molecules, including membrane ruffles, folds or protrusions, making this a widespread problem for the quantification of local membrane concentration.

It is noteworthy that despite similar asymmetry of anterior structures, only CDC-42, which is known to interact with anterior-enriched PAR proteins, exhibited pronounced polarity when quantified in cross-section and retained this asymmetric enrichment during the maintenance phase when filopodia largely disappeared. These data argue against overall asymmetry of either PIP_2_ or RHO-1 or for filopodia being required for CDC-42 asymmetry (Fig. S4). One should also note that the localization of activity sensors for CDC-42 and RHO-1 tend not to match localization of the proteins overall (Nishikawa et al., 2017; Kumfer et al., 2010), consistent with local regulation of activity, rather than local accumulation alone, being critical for localized function of these GTPases. Anterior PIP_2_ enrichment is also difficult to reconcile with observations that the PI4K kinase, PPK-1, is modestly enriched in the embryo posterior, which is opposite to what would be expected if high PIP_2_ levels defined the anterior (Panbianco et al., 2008). LGL and PAR-2 are also both thought to rely on PIP_2_ for membrane association, despite being enriched in the posterior (Motegi et al., 2011; Dong et al., 2015). We therefore favor a global, rather than local, role for PIP_2_, which is consistent with the sensitivity of the zygote to bulk changes in PIP_2_ levels (Scholze et al., 2018).

The existence of PIP_2_ membrane domains remains controversial (van Rheenen and Jalink, 2002; Stone et al., 2017; van den Bogaart et al., 2011; Wang and Richards, 2012; Ji et al., 2015). While we cannot rule out the existence of PIP_2_ membrane domains that are not revealed by the probes used to date, in light of our data, we feel there is currently no compelling experimental evidence to support the existence of PIP_2_ microdomains or anterior PIP_2_ enrichment in the *C. elegans* zygote.

## MATERIALS AND METHODS

### Strains, growth and media

*C. elegans* strains were maintained on nematode growth medium (NGM) under standard conditions (Brenner, 1974) at 16°C or 20°C unless otherwise indicated. Strains are listed in Table S1.

#### Strain construction

mKate_myr_ consists of the first 11 amino acids of SRC-2, harboring the N-myristoylation site, followed by a 3×Myc tag, mKate and the coding sequence of iLID (Guntas et al., 2015). The coding sequence is expressed under the *mex-5* promoter and *nmy-2* 3′UTR in plasmid pNG17, which was introduced by biolistic bombardment into DP38 worms creating strain NWG0045 (Praitis et al., 2001). SWG19 was generated by backcrossing SWG4 (Reymann et al., 2016) to the N2 strain (four times). For membrane tethering of LifeAct::GFP, we crossed NWG0047 (PH::GBP::mKate) with TH220 (LifeAct::GFP).

#### RNAi

RNAi was performed according to previously described methods (Kamath et al., 2003). Briefly, HT115(DE3) bacterial feeding clones were inoculated from LB agar plates to LB liquid cultures and grown overnight at 37°C in the presence of 10 µg/ml carbenicillin. 100 µl of bacterial cultures was spotted onto 60 mm agar RNAi plates (10 µg/ml carbenicillin, 1 mM IPTG). L4 larva were added to RNAi feeding plates and incubated for 20–48 h depending on gene and temperature. RNAi clones targeting *arx-2*, *ect-2*, *par-2*, *perm-1*, *pkc-3* and *wve-1* were obtained from the Ahringer library, which is currently available via Source BioScience (Nottingham, UK).

#### Embryo dissection and mounting

For imaging, embryos were typically dissected in egg buffer (118 mM NaCl, 48 mM KCl, 2 mM CaCl_2_, 2 mM MgCl_2_, 25 mM HEPES pH 7.4) or M9 buffer, and mounted under a 2% or 3% agarose pad and sealed with VALAP (1:1:1, Vaseline, lanolin and paraffin wax). For FM4-64 experiments, *perm-1(RNAi)* embryos were dissected and mounted in 0.75% egg buffer, with 18–20 µm beads (Polysciences, Warrington, PA) under a coverslip, and two edges were sealed with VALAP to create a flow chamber (Carvalho et al., 2011; Goehring et al., 2011). FM4-64 (T13320, ThermoFisher UK, 5 µg/ml in 0.75% egg buffer) was then introduced by capillary action.

### Microscopy and image acquisition

#### Confocal image acquisition

Midsection images were captured on a Nikon TiE with a 100×1.45 NA objective, further equipped with a custom X-Light V1 spinning disk system (CrestOptics, Rome, Italy) with 50 µm slits, Obis 488/561 fiber-coupled diode lasers (Coherent, Santa Clara, CA) and an Evolve Delta EMCCD camera (Photometrics, Tuscon, AZ). Imaging systems were run using Metamorph (Molecular Devices, San Jose, CA) and configured by Cairn Research (Kent, UK). Filter sets were from Chroma (Bellows Falls, VT): ZT488/561rpc, ZET405/488/561/640X, ET525/50m, ET630/75m and ET655LP.

Surface confocal images were acquired with spinning disk confocal microscope every 2 s [for CYK-1: Zeiss C-Apochromat with a Yokogawa CSU-X1 scan head, Orca-Flash4.0 camera (Hamamatsu Photonics, Japan) and a 100×/1.42 NA objective lens, run using Micro-Manager; for PLST-1: Inverted Nikon Eclipse Ti equipped with a Yokogawa CSU-X1 scan head, simultaneous dual camera with two Prime 95B cameras (Photometrics) and a 100×1.4 NA objective lens, configured by Gataca Systems (Massy, France) and run using Metamorph].

#### HiLo imaging

Unless otherwise specified, surface images were captured by HiLo microscopy (Konopka and Bednarek, 2008; Tokunaga et al., 2008) on a Nikon TiE with a 100× N.A. 1.49 objective, further equipped with a iLAS TIRF unit (Roper, Lisse, France), custom field stop, Obis 488/561 fiber-coupled diode lasers (Coherent) and an Evolve Delta camera. Imaging systems were run using Metamorph and configured by Cairn Research. Filter sets were from Chroma: ZT488/561rpc, ZET488/561x, ZET488/561m, ET525/50m, ET630/75m, ET655LP. FRAP was performed in a 6.2×6.2 µm box in the anterior of maintenance phase embryos with 20 prebleach frames and an imaging interval of 0.5 s.

### Data analysis

Image processing and data analysis were performed in Python (www.python.org), Matlab (Mathworks, Natick, MA) and Fiji (Schindelin et al., 2012). For statistical comparisons, all data points are shown and significance assessed using a Student's *t*-test, two-tailed.

#### FRAP

FRAP analysis was performed in Matlab using scripts provided in Goehring et al. (2010), but fit to a single exponential to extract *τ*_1/2_.

#### CYK-1 tracking

Filopodia tip velocity measurements were obtained by tracking CYK-1::GFP puncta, which was performed in Python using the ‘trackpy’ package (https://github.com/soft-matter/trackpy). Custom Python code developed for the analysis is available at https://github.com/lhcgeneva/SPT. Briefly, a Crocker–Grier algorithm detects local intensity peaks, which are then fit to a Gaussian point spread function with the detection threshold adjusted empirically for imaging conditions. An independently acquired dataset was quantified using the MOSAIC plugin in Fiji (http://mosaic.mpi-cbg.de/?q=downloads/imageJ) together with custom Matlab codes (available from corresponding author upon request) for data analysis to confirm results.

#### Spatial/temporal fluorescence profiles

In general, fluorescence profiles (both experimental and simulated) were obtained by tracking a 3-pixel-wide line along the membrane from images subjected to a Gaussian Blur (*σ*=1 px) to reduce noise. Mean normalized profiles after subtraction of chip background were extracted and plotted in Matlab.

For Fig. 3G, clear filopodia-like structures were identified that were isolated from other structures that would complicate analysis. After obtaining fluorescence profiles along filopodia in both channels, data from each filopodium was aligned based on the peak of GFP::LifeAct intensity.

For Fig. 3H, fluorescence profiles along the path of the actin comets were obtained over time, and the data plotted as a two-channel kymograph. Temporal change was calculated across a minimum of ten spatial positions for each individual comet, the resulting data aligned by the time of peak GFP fluorescence, before averaging to obtain the average temporal profile of GFP and mKate for each comet. Δτ was defined as the peak-to-peak time difference between maximal GFP and mKate accumulation calculated from average temporal profiles of each comet.

For Fig. 3K, profiles of LifeAct::mKate and PH_PLCδ1_ relative to CYK-1 puncta were obtained by first identifying clear filopodia with comet-like morphologies from a minimum of three embryos each. A 3-pixel line beginning at the center of the CYK-1 focus and running through the PH- or LifeAct-labeled region was then defined and straightened in Fiji. Fluorescence profiles were then extracted in Matlab, normalized to the mean intensity and plotted as a function of distance from CYK-1 puncta at the filopodia tip.

For quantification of relative peak intensities in Figs 1I and 4F, 3-pixel-wide profiles across membrane features were extracted; then, cytoplasmic background was subtracted, and the top three peak intensity pixels summed. Data was normalized to median intensities obtained in regions of the plasma membrane devoid of membrane structures, representing a single bilayer configuration, in the same embryo. Simulated images were treated identically except that they were normalized to the median value of all single membrane peaks.

#### Colocalization

Regions of interest (ROIs) were manually defined for a minimum of 30 well-defined and separated structures in the reference channel for each embryo, usually using the channel showing fluorescent protein fusions to PH_PLCδ1_. ROIs were then queried in the test channel to score whether the structure was labeled by the other molecule, scoring either for the presence of a similar structure or a tip-localized punctum, in the case of CYK-1. The fraction of structures showing colocalization was calculated for each embryo.

#### Asymmetry index

For Fig. 3C, the asymmetry index (ASI) of cortical LifeAct was calculated by first obtaining mean fluorescence values from selected regions of the cell cortex in the anterior and posterior halves of the zygote in background subtracted images. We then calculated ASI according to the equation ASI=(A−P)/[2(A+P)], where A and P are the fluorescence values in the anterior and posterior, respectively. The resulting values for ASI range from −0.5 to 0.5, with 0 being symmetric, and −0.5 and 0.5 being maximally polarized towards posterior or anterior, respectively.

In Fig. S4, the ASI was calculated from membrane intensity profiles around the circumference of the embryo extracted from cross-sectional confocal images. Briefly, a 50-pixel-wide line following the membrane around the embryo was computationally straightened, and a normalized cytoplasmic GFP curve was subtracted to isolate membrane signal following the procedure described in Reich et al. (2019). Mean intensity values corresponding to the posterior and anterior regions of the embryo (each representing one-third of the total circumference) were then used to calculate ASI as above.

#### Image simulations

To simulate fluorescence microscopy images of hypothesized experimental membrane configurations, a starting image of resolution 5 nm/pixel was generated to match the dimensions of the experimental image in Fig. 4B. The membrane bilayer was simulated as a 1-pixel-wide line, which was used to trace the hypothesized membrane configuration from the experimental image. This included a region containing part of the pseudocleavage furrow, which generates a double membrane as well as a circle 100 nm in diameter to mimic the cross section of the filopodial membrane. A uniform background level of photons was added before subjecting the resulting image to a 200-nm-wide Guassian blur and resampling to the experimental resolution of 0.155 µm/pixel. Modulated Poisson noise and readout noise (five standard deviations) was then added before processing identically to the experimental image. All manipulations were performed in Fiji.

## Supplementary Material

Supplementary information
