# Supplementary material for: Anterior-enriched filopodia create the appearance of asymmetric membrane microdomains in polarizing C. elegans zygotes
Source: J Cell Sci. 2019 Jul 15;132(14):jcs230714. doi: 10.1242/jcs.230714 (PMC6679585; doi:10.1242/jcs.230714)
Supplement: Supplementary information [file joces-132-230714-s1.pdf]

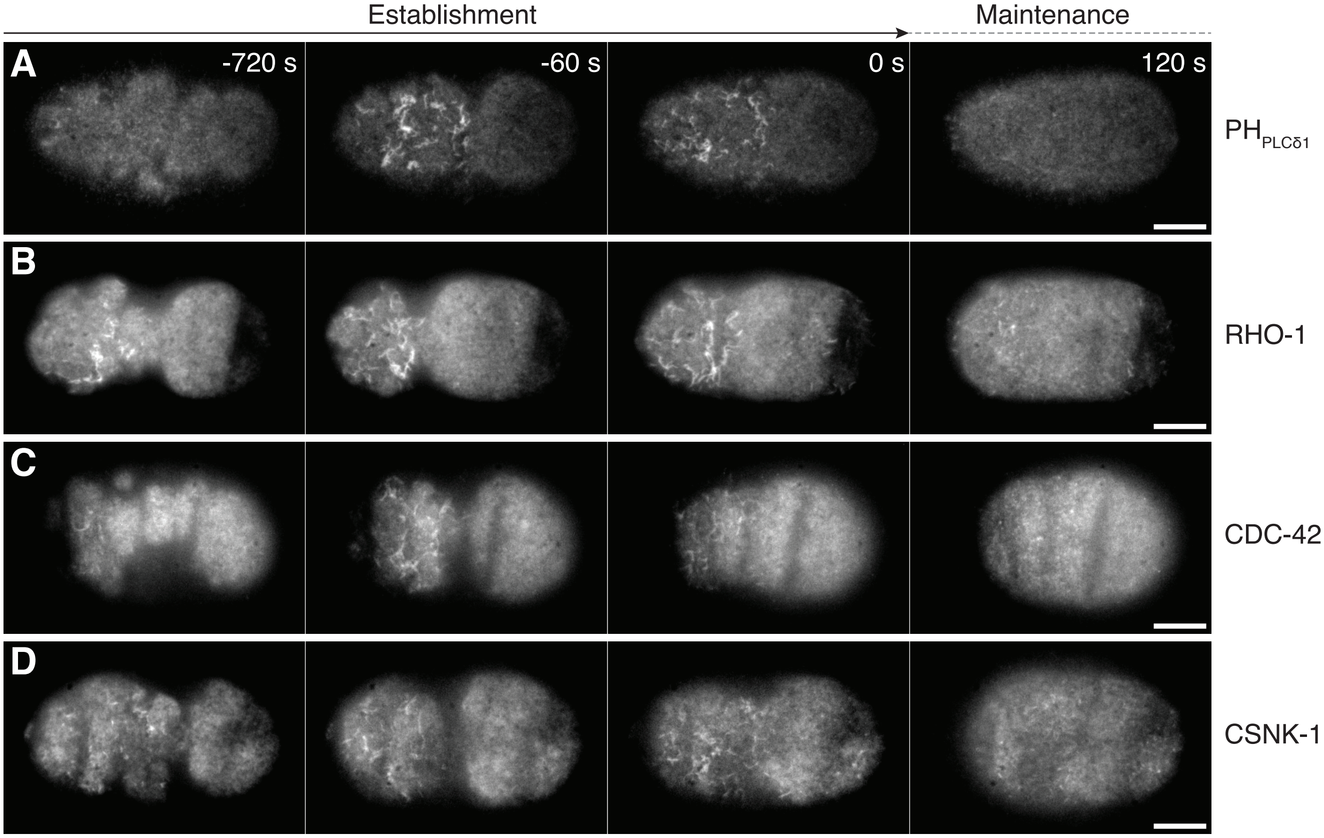


**Figure S1. Diverse signaling molecules appear enriched in cortical structures. (A-D)** HiLo microscopy images of mCherry::PH$_{PLC\delta 1}$ (n>5) (A), GFP::RHO-1 (n = 2) (B), CDC-42::mCherry (n = 3) (C), and GFP::CSNK-1 (n = 2) (D) at the cell cortex are shown from early symmetry-breaking to maintenance phase. All show cortical structures with a distinct pattern of appearance, subsequent enrichment in the anterior and dissipation upon entry to maintenance phase. Time (s) relative to the end of establishment phase. Scale bar = 10 um.

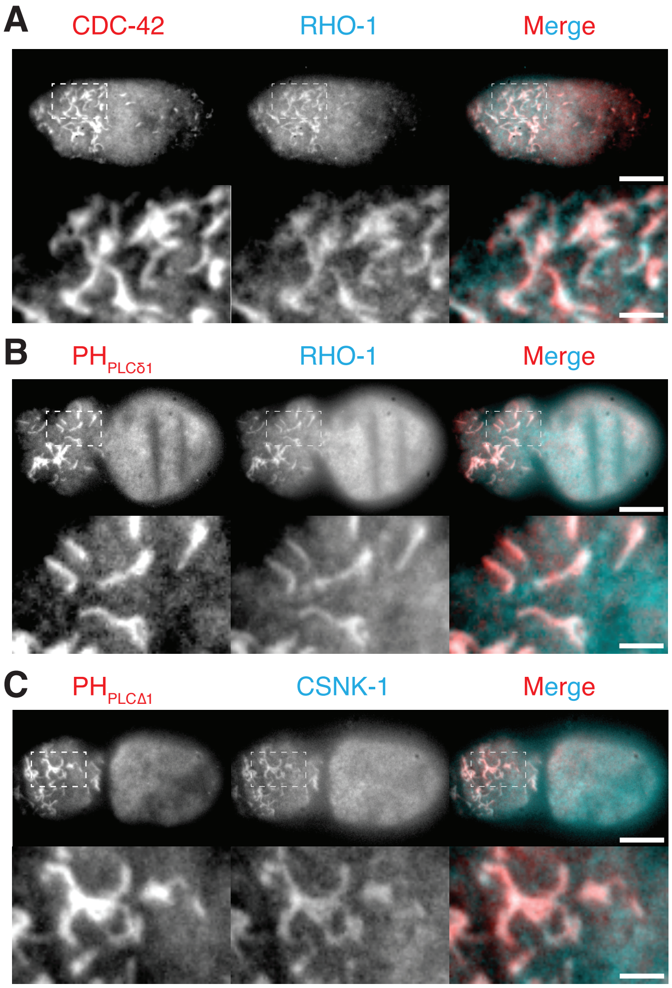


**Figure S2. Colocalization of signaling molecules with the PIP$_2$ probe PH$_{PLC\delta1}$. (A)** Sample image of surface of embryo co-expressing GFP::RHO-1 and mCherry CDC-42. Individual channels and merge shown with zoom of inset region to highlight structures. **(B)** As in (A), but GFP::RHO-1 with mCherry::PH$_{PLC\delta1}$. **(C)** As in (A), but GFP::CSNK-1 with mCherry::PH$_{PLC\delta1}$. Co-enrichment quantified in Figure 1B. Scale bars = 10 um.

Journal of Cell Science • Supplementary information

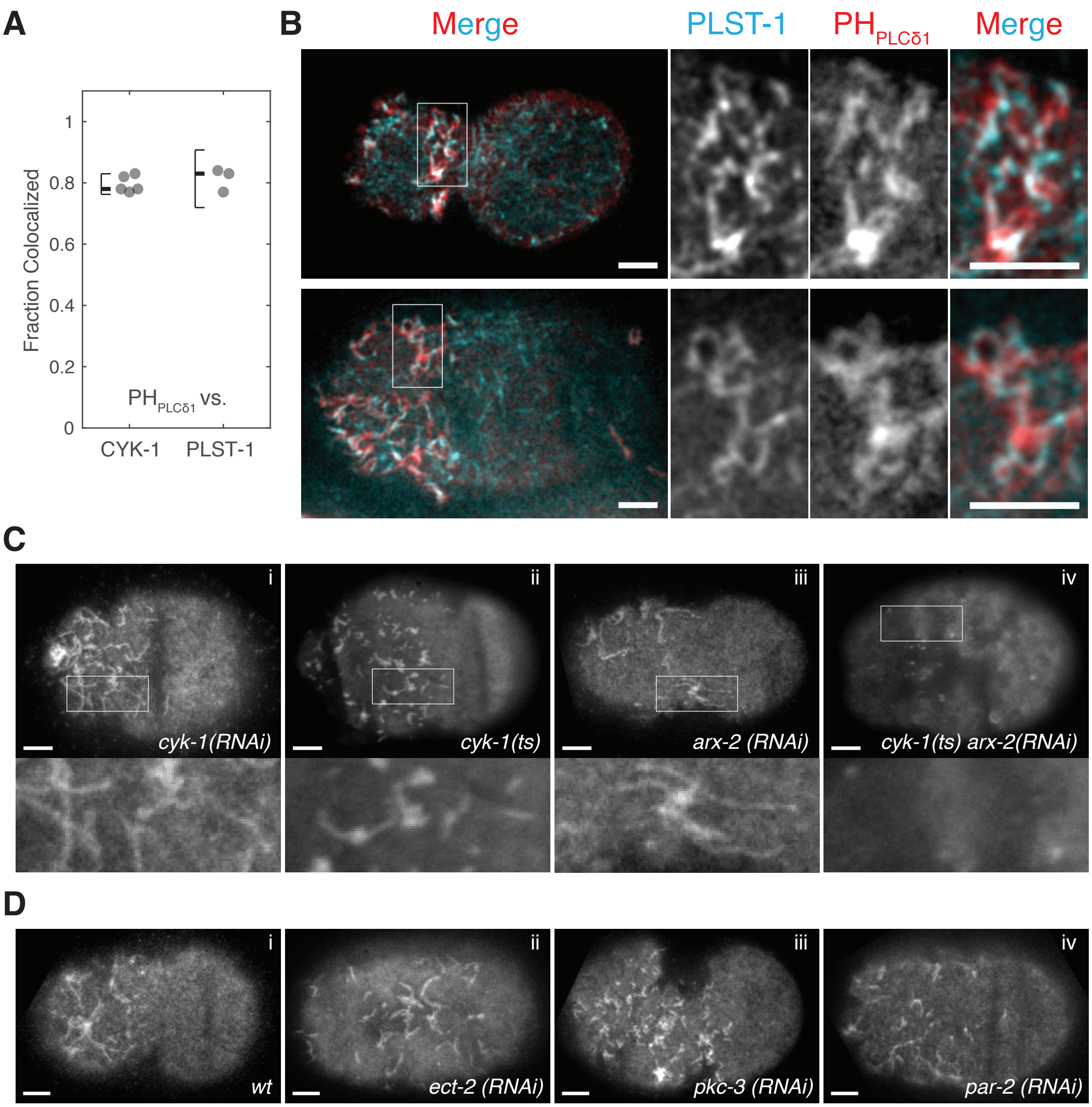


**Figure S3. Filopodia are enriched in actin bundling proteins, require actin polymerization, and respond to polarity cues.** **(A)** Fraction of selected PH$_{PLC\delta1}$-labeled structures that exhibit colocalized CYK-1 puncta at tips or enrichment in PLST-1. Datapoints from individual embryos shown. Median ± 95%CI indicated. **(B)** Cortical images of two embryos expressing PLST-1::GFP and mCherry::PH$_{PLC\delta1}$. Inset is marked and magnified on right showing merged and single channel images. **(C)** Disruption of both CYK-1 and ARX-2 lead to loss of filopodia like structures. mCherry (i,iii) or GFP (ii,iv) fusions to PH$_{PLC\delta1}$ persist when either ARX-2 or CYK-1 are disrupted on their own, but not when both are compromised. Insets magnified below to highlight filopodia structure. **(D)** Formation of filopodia is not affected when contractility is compromised by depletion of the RhoGEF, ECT-2, or when polarity is disrupted by depletion of PKC-3 or PAR-2 by RNAi. However, asymmetry of filopodia is reduced in all three cases consistent with their asymmetry requiring polarity. ECT-2 depletion compromises symmetry-breaking during the establishment phase (***Zonies et al., 2010***). Images shown reflect a timepoint of peak filopodial density just before relaxation of the cortex and filopodial disassembly. Scale bars = 5 $\mu$m.

Journal of Cell Science • Supplementary information

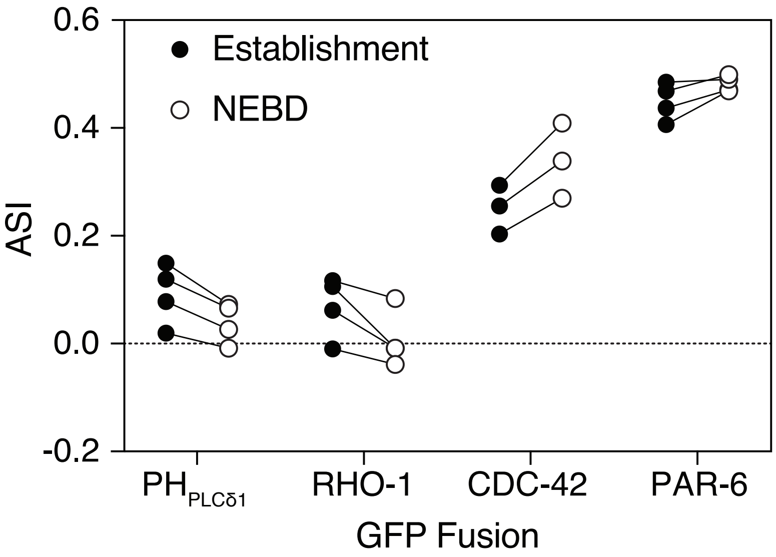


**Figure S4. Asymmetry in membrane concentration from midplane images.** Membrane fluorescence profiles were traced from midplane images of embryos expressing the indicated GFP fusions at mid-establishment (5 min pre-NEBD) or mid-maintenance phase (NEBD). For each embryo the asymmetry index (ASI) was calculated at each timepoint from the extracted profiles. Note asymmetry of both $PH_{PLC\delta1}$ and RHO-1 is low at establishment phase and is reduced further at maintenance phase when filopodia dissipate, consistent with filopodia contributing to weak apparent asymmetry during the establishment phase. By contrast, CDC-42 exhibits moderate asymmetry during establishment when anterior filopodia are high, but its asymmetry increases further by NEBD, when filopodia dissipate. For reference, the polarity protein PAR-6, which is not prominently enriched in these structures due to association with PAR-3 clusters, is highly polarized at both time points. Together these data suggest that filopodia-like structures, despite being highly asymmetric, do not contribute significantly to overall polarity in membrane concentrations.

Journal of Cell Science • Supplementary information

**Table S1.** *C. elegans* strains used in this work.

| Strain | Genotype | Source |
|---|---|---|
| ACR004 | *cyk-1(ges1[cyk-1::eGFP + LoxP unc-119(+) LoxP] III; unc-119 (ed3) III; ltIs44pAA173; [pie-1p::mCherry::PH(PLC1delta1) + unc-119(+)] V* | This work |
| ACR010 | *plst-1(ges2[plst-1::eGFP + LoxP unc-119(+) LoxP] IV; unc-119 (ed3) III; ltIs44pAA173; [pie-1p::mCherry::PH(PLCδ1) + unc-119(+)] V* | This work |
| AD189 | *unc-119(ed3) III; asIs2[unc-119(+) + pie-1p::GFP::egg-1]* | CGC, **Kadandale et al.** (**2005**) |
| FT204 | *unc-119(ed3) III; xnIs87[syn-4p::GFP::syn-4::syn-4 3'UTR + unc-119(+)]* | CGC |
| JA1354 | *unc-119(e2498) III; weIs12[unc-119(+) + pie-1p::GFP::csnk-1]* | CGC, **Panbianco et al.** (**2008**) |
| JCC146 | *cyk-1(or596ts); unc-119(ed3); ltIs38 [pAA1; pie-1p::GFP::PH(PLCδ1) + unc-119 (+)]; ltIs37 [pAA64; pie-1p::mCherry::his-58; unc-119 (+)]IV* | **Jordan et al.** (**2016**) |
| KK1248 | *par-6(it310[par-6::gfp]) I* | CGC, Ken Kemphues |
| NWG0045 | *unc-119(ed3) III; crkIs16[mex-5p::mKate::iLiD::nmy-2 3'UTR + unc-119(+)]* | This work |
| NWG0047 | *unc-119(ed3)III; crkEx1[pNG19: mex-5p::PH(PLCδ1)::GBP::mKate::nmy- 2 3'UTR + unc-119(+)]; him-5 (e1490) V* | **Rodriguez et al.** (**2017**) |
| OD58 | *unc-119(ed3) III; ltIs38[pAA1; pie-1p::GFP::PH(PLCδ1) + unc-119(+)]* | CGC, **Audhya et al.** (**2005**) |
| OD70 | *unc-119(ed3) III; ltIs44pAA173; [pie-1p-mCherry::PH(PLCδ1) + unc-119(+)] V* | CGC, **Kachur et al.** (**2008**) |
| SA115 | *tjIs1[pie-1::GFP::rho-1 + unc-119(+)]* | CGC, **Motegi and Sugimoto** (**2006**) |
| SWG1 | *mex-5p::Lifeact::mKate2* | **Reymann et al.** (**2016**) |
| SWG5 | *plst-1(ges2[plst-1::eGFP + LoxP unc-119 (+) LoxP] IV; unc-119 (ed3) III)* | **Reymann et al.** (**2016**) |
| SWG19 | *cyk-1(ges1[cyk-1::eGFP + LoxP unc-119(+) LoxP) III; unc-119(ed3) III.* | **Reymann et al.** (**2016**) |
| TH159 | *ddIs46[WRM0625bA11 GLCherry::cdc-42; Cbr-unc-119(+)]* | **Rodriguez et al.** (**2017**) |
| TH220 | *unc-119(ed3) III; ddIs86[pie-1p::LifeAct::GFP; unc-119(+)]* | **Redemann et al.** (**2010**) |
| WS5018 | *cdc-42(gk388) opIs295[cdc-42p::GFP::cdc-42(genomic)::cdc-42 3'UTR + unc-119(+)] II.* | CGC, **Neukomm et al.** (**2014**) |

Journal of Cell Science • Supplementary information

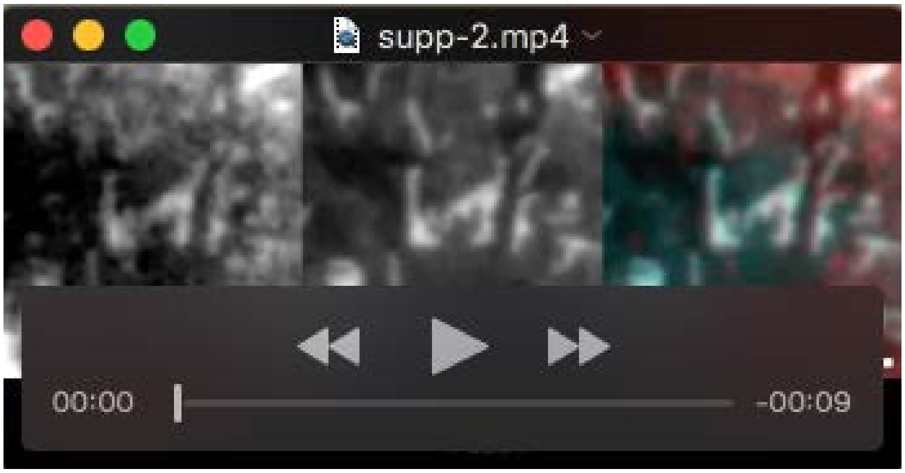


**Movie 1.** Timelapse video of the surface of a permeabilized embryo expressing $PH_{PLC\delta1}$::GFP (middle, cyan) and stained with FM4-64 (left, red). Scale bar, 2.5 µm. Elapsed time (sec). See related Figure 1F.

Journal of Cell Science • Supplementary information

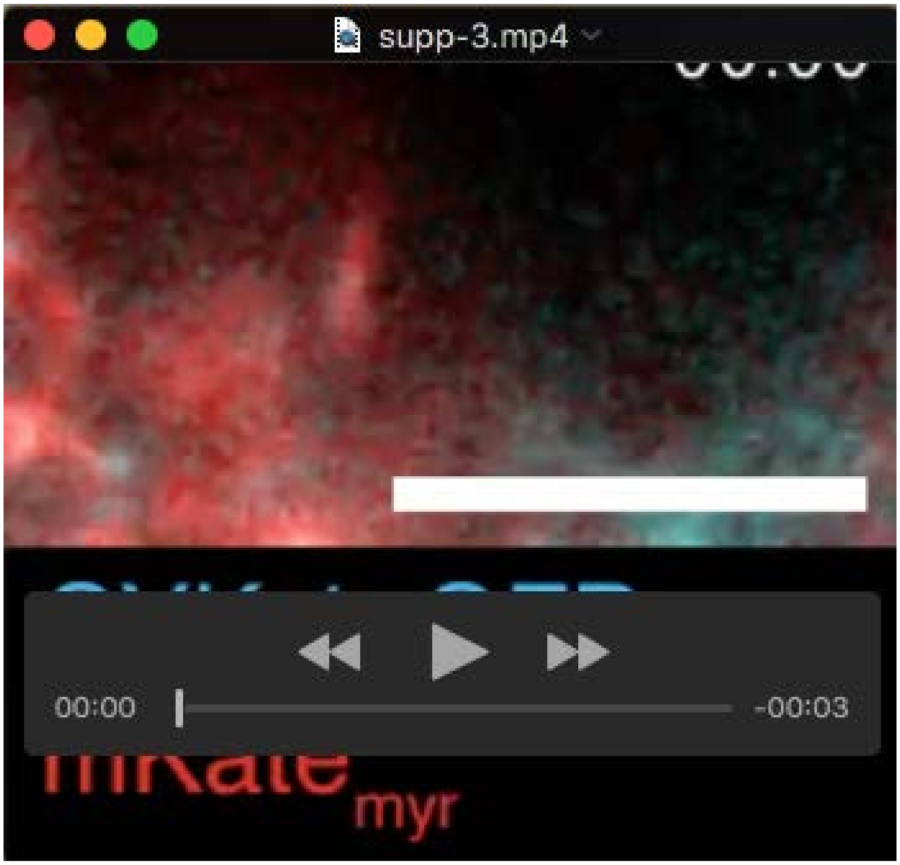


**Movie 2.** Timelapse video of filopodia extending into the pseudocleavage furrow in an embryo expressing CYK-1::GFP and $\text{mKate}_{\text{myr}}$. Scale bar, 5 µm. Elapsed time (mm:ss) shown. See related Figure 2A.

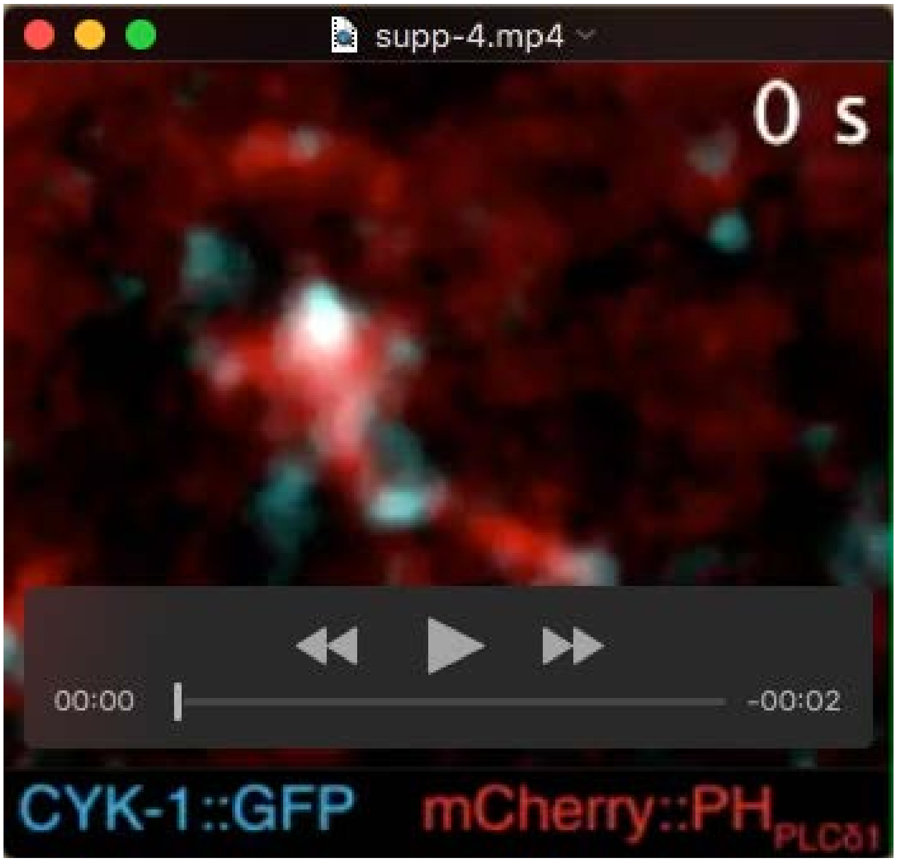


**Movie 3.** Timelapse video of dynamic filopodia on the surface of an embryo expressing CYK-1::GFP and $_{PLC\delta1}$::mCherry. Scale bar, 5 µm. Elapsed time (sec) shown. See related Figure 2D.

Journal of Cell Science • Supplementary information

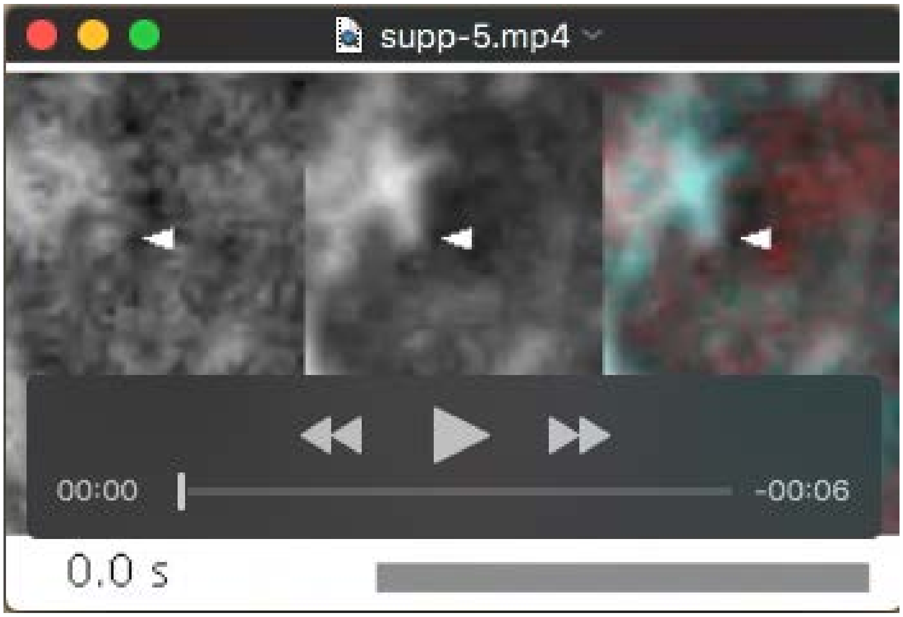


**Movie 4.** Timelapse video of an extending filopodium labeled with LifeAct::GFP and $_{PLC\delta1}$::mCherry on the surface of a one-cell embryo. Elapsed time (sec) shown. Scale bar, 5 µm. See related Figure 2F.

Journal of Cell Science • Supplementary information

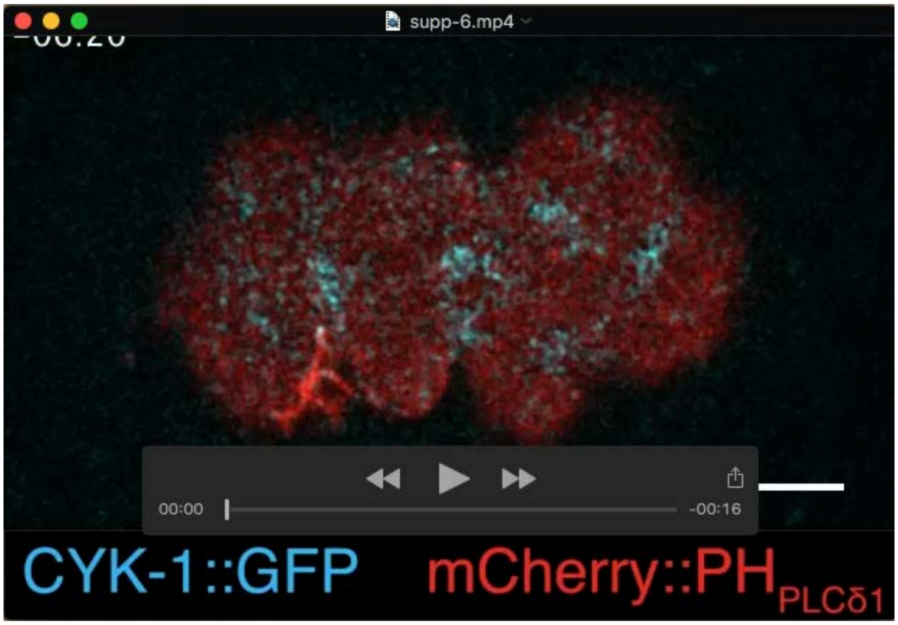


**Movie 5.** Timelapse video of the surface of an embryo expressing CYK-1::GFP and $PH_{PLC\delta1}$::mCherry by confocal microscopy showing the appearance, accumulation, segregation and dissipation of filopodia. Elapsed time shown (mm:ss). Scale bar, 5 µm. See related Figure 2K.

Journal of Cell Science • Supplementary information

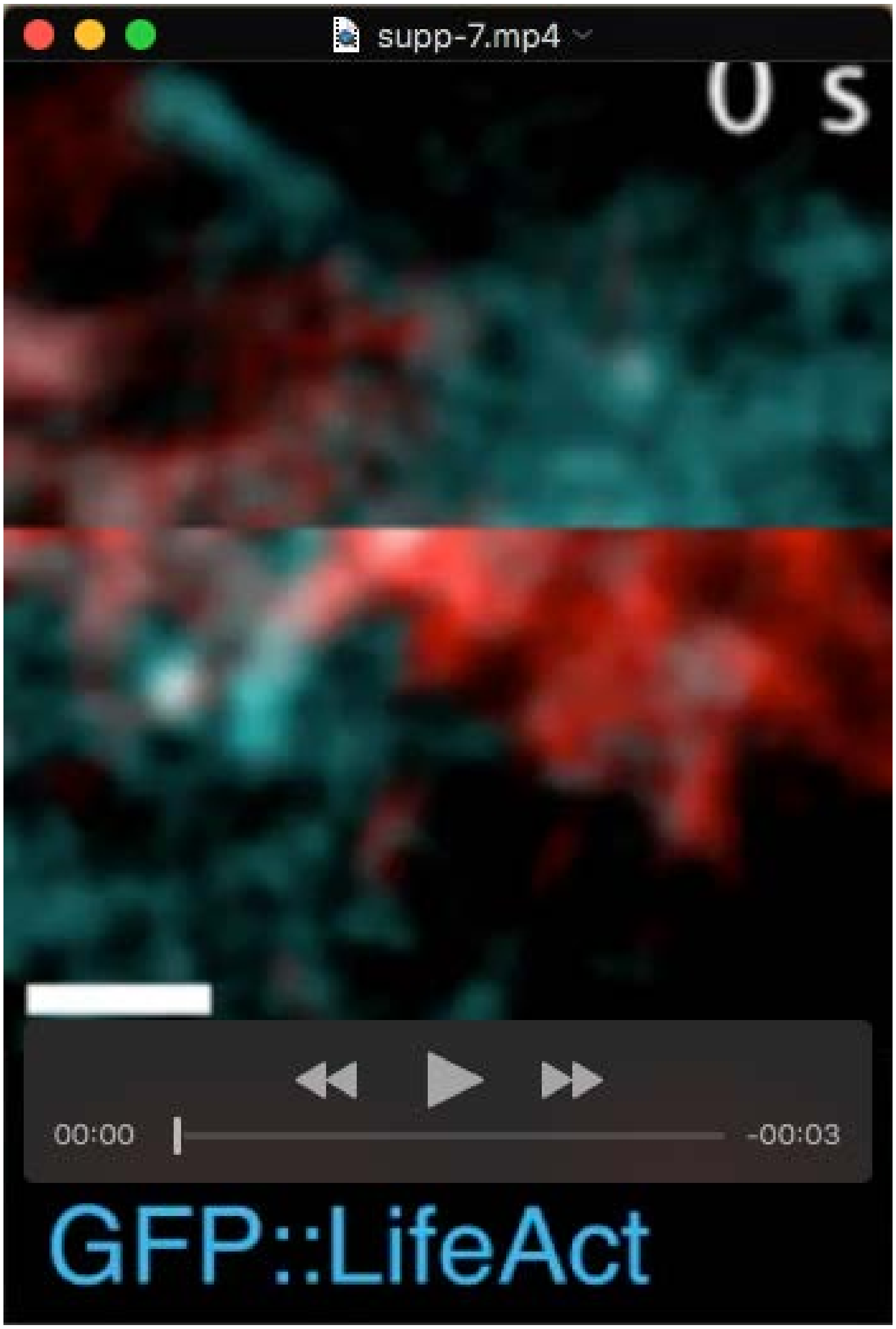


**Movie 6.** Timelapse video of two filopodia labeled with LifeAct::mKate (red) and LifeAct::GFP (cyan) corresponding to straightened filopodia shown in Figure 3E. Note lag of LifeAct::mKate signal relative to LifeAct::GFP yielding a cyan tip followed by a red tail. Elapsed time shown (sec). Scale bar, 2.5 µm. See related Figure 3F-G.

Journal of Cell Science • Supplementary information

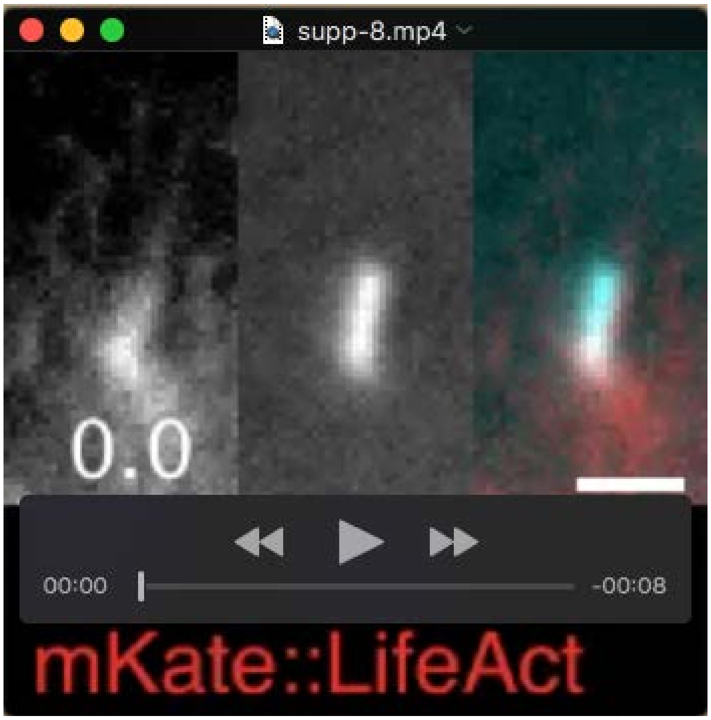


**Movie 7.** Timelapse video of cytoplasmic actin comet labeled with LifeAct::mKate (red) and LifeAct::GFP (cyan). Note lag of LifeAct::mKate signal relative to LifeAct::GFP yielding a cyan tip followed by a red tail. Elapsed time shown (sec). Scale bar, 2.5 µm. See related Figure 3G-H.

Journal of Cell Science • Supplementary information

## References

**Audhya A., Hyndman F., McLeod I.X., Maddox A.S., Yates J.R., Desai A., Oegema K.** (2005). A complex containing the Sm protein CAR-1 and the RNA helicase CGH-1 is required for embryonic cytokinesis in *Caenorhabditis elegans*. *J. Cell Biol.* 171, 267–279.

**Cuenca A.A., Schetter A., Aceto D., Kemphues K., Seydoux G.** (2003). Polarization of the *C. elegans* zygote proceeds via distinct establishment and maintenance phases. *Development* 130, 1255–1265.

**Jordan S.N., Davies T., Zhuravlev Y., Dumont J., Shirasu-Hiza M., Canman J.C.** (2016). Cortical PAR polarity proteins promote robust cytokinesis during asymmetric cell division. *J. Cell Biol.* 212, 39–49.

**Kachur T.M., Audhya A., Pilgrim D.B.** (2008). UNC-45 is required for NMY-2 contractile function in early embryonic polarity establishment and germline cellularization in *C. elegans*. *Dev. Biol.* 314, 287–299.

**Neukomm L.J., Zeng S., Frei A.P., Huegli P.A., Hengartner M.O.** (2014). Small GTPase CDC-42 promotes apoptotic cell corpse clearance in response to PAT-2 and CED-1 in *C. elegans*. *Cell Death Differ.* 21, 845–853.

**Redemann S., Pecreaux J., Goehring N.W., Khairy K., Stelzer E.H.K., Hyman A.A., Howard J.** (2010). Membrane Invaginations Reveal Cortical Sites that Pull on Mitotic Spindles in One-Cell *C. elegans* Embryos. *PLoS ONE.* 5, e12301.

**Zonies S., Motegi F., Hao Y., Seydoux G.** (2010). Symmetry breaking and polarization of the *C. elegans* zygote by the polarity protein PAR-2. *Development* 137, 1669–1677.

Journal of Cell Science • Supplementary information
